# Alteration of Serum Free Fatty Acids are Indicators for Progression of Pre-leukaemia Diseases to Leukaemia

**DOI:** 10.1038/s41598-018-33224-1

**Published:** 2018-10-05

**Authors:** Ayesha Khalid, Amna Jabbar Siddiqui, Jian-Hua Huang, Tahir Shamsi, Syed Ghulam Musharraf

**Affiliations:** 1 H.E.J. Research Institute of Chemistry, International Center for Chemical and Biological Sciences, University of Karachi, Karachi, 75270 Pakistan; 2 Dr. Panjwani Center for Molecular Medicine and Drug Research, International Center for Chemical and Biological Sciences, University of Karachi, Karachi, 75270 Pakistan; 3 National Institute of Blood Diseases and Bone Marrow Transplantation, Karachi, Pakistan; 4 TCM and Ethnomedicine Innovation and Development Laboratory, Changsha, Hunan China

## Abstract

Acute Leukaemia (AL) is a neoplasm of WBCs (white blood cells). Being an important class of metabolites, alteration in free fatty acids (FFAs) levels play a key role in cancer development and progression. As they involve in cell signaling, maintain membrane integrity, regulate homeostasis and effect cell and tissue functions. Considering this fact, a comprehensive analysis of FFAs was conducted to monitor their alteration in AL, pre-leukaemic diseases and healthy control. Fifteen FFAs were analyzed in 179 serum samples of myelodysplastic syndrome (MDS), aplastic anemia (APA), acute lymphoblastic leukaemia (ALL), acute myeloid leukaemia (AML) and healthy control using gas chromatography-multiple reaction monitoring-mass spectrometry (GC-MRM-MS). A multivariate statistical method of random forest (RF) was employed for chemometric analysis. Serum level of two FFAs including C18:0 and C14:0 were found discriminative among all five groups, and between ALL and AML, respectively. Moreover, C14:0 was identified as differentiated FFAs for systematic progression of pre-leukaemic conditions towards AML. C16:0 came as discriminated FFAs between APA and MDS/AML. Over all it was identified that FFAs profile not only become altered in leukaemia but also in pre-leukaemic diseases.

## Introduction

Among four basic macromolecules i.e. carbohydrates, nucleic acids, proteins and lipids found in human blood, lipids are the most diversified in their structure. Lipids have various distinct molecular species due to which lipids have gained attention like other three macromolecules. Eight categories in which lipids are divided, FFAs are the most important molecular constituents and have diverse functions that not only encompass as an energy source and part of cell membrane but also have other countless biological activities by which FFAs have an impact on health, wellbeing and disease risk^1^. FFAs synthesis in the body promotes tumorigenesis and tumor progression, one of the main reason that it has received ample attention as a potential target for cancer diagnosis, progression, and treatment over the past few years^2–4^. It was observed that breast cancer cells of human express an elevated expression and activity level of fatty acid synthase (FAS). After several basic and clinical research, it can be stated that cancer cells have an ability to produce their own fatty acid (FA) supply, that is apparently free from the regulatory signaling pathways that down-regulate FA synthesis in healthy cells^5^. Prior studies show the endogenous FAs production might be a major supply of FAs for development of cancer cells^5,6^. Links have also been found between the increased concentration of particular FAs in responses to osteoarthritis, breast cancer incidence, arterial stiffness, type 2 diabetes, cardiovascular risk and schizophrenia^7–12^.

Leukaemia, among the 10^th^ foremost roots of cancer-related decease throughout the world, can be subdivided into ALL and AML^13^. Among all cancers that diagnosed in kids younger than 15 years, AL shares 30% of this cancer. Surrounded by this count, ALL in comparison to AML befalls around five times more frequent and share about more than three-quarters of total childhood leukaemia cases diagnose^14,15^. In the light of 2012 Globocan report, each year approximately the reported fresh cases of leukaemia were 0.35 million and the mortality rate in adults and children due to leukaemia was 74%^16^. Of all AML cases, 80% reported in adults. For leukaemia, the survival rate of 5-year is 54%. Though, comparative survival rates over 5-year for below age 15 children are found to be 60% for AML and 89% for ALL^17^.

It has been observed that 10–30% of the survived patients from APA or MDS would eventually develop leukeamia mainly AML in late stages of their life. However, to our knowledge, there is no correlative biomarker available which monitor the progression of pre-leukaemic conditions towards AML. In a previous untargeted metabolomics study on AL it was reported that majority of discriminating metabolites identified in AL were lipids in comparison with controls^18^. However, there are few lipidomics studies on acute leukaemia^19,20^. To date, there have been no studies on quantification of FFAs profiles of ALL, AML and pre-leukaemic conditions that are APA and MDS to exploit any distinctive FFA that may serve as differentiated biomarker among these groups. In the present study, we have analyzed FFAs profiles in AL patients (AML and ALL), pre-leukaemic (APA, MDS) and healthy subjects using GC-MS followed by chemometric analysis using RF algorithm that serve as a new and powerful classifying model that not only use to discriminate groups but also provide useful information about distinguishing metabolites that may serve as biomarkers.

## Experimental

### Ethical statement

This study was approved by the ethical committee of National Institute of Blood Diseases and Bone Marrow Transplantation (NIBD), and by the Independent Ethic Committee (IEC) of International Center of Chemical and Biological Sciences (ICCBS), Karachi, Pakistan. All samples were collected, stored and processed according to the approved protocol. A written informed consent was obtained from all patients and healthy individuals.

### Sample collection

Overall 24 cases were registered with ALL, 46 cases with APA, 62 cases with AML, and 17 cases with MDS. It was taken into account that before sample collection patients were neither in remission stage nor they received any sort of therapy. Patients transformed from MDS or leukaemia secondary to other malignancies were not encompassed in this investigation. 30 healthy controls that are equivalent to their ages and gender were also enlisted as negative controls in order to eliminate any age and gender effects. Subdivision of AML and ALL are stated in the Table S1. After overnight fasting, serum samples were collected from the patients (for minimum 10 h) in BD Vacutainer tubes (BD Franklin Lakes, NJ, USA, REF 367381), as clot activator silicone coated interiorly. After half an hour, serum was parted by centrifugation at 2000 rpm at 4 °C for 10 min. After that, the aliquots of sera were made and kept in −80 °C freezer until further analysis was performed.

### Solvents and standards

Purchasing of all fatty acid methyl ester (FAME) standards including methyl octanoate (C-8:0), methyl decanoate (C-10:0), methyl dodecanoate (C-12:0), methyl tetradecanoate (C-14:0), methyl hexadecanoate (C-16:0), methyl octadecanoate (C-18:0), methyl docosanoate (C-22:0), methyl tetracosanoate (C-24:0), methyl-cis-9-tetradecenoate (C-14:1 Δ^cis−9^), methyl-cis-9-hexadecenoate (C-16:1 Δ^cis−9^), methyl-cis-9-octadecenoate (C-18:1 Δ^cis−9^), methyl-cis-11-octadecenoate (C-18:1 Δ^cis−11^), methyl-cis-11-eicosenoate (C-20:1 Δ^cis−11^), methyl-cis-13-docosenoate (C-22:1 Δ^cis−13^) and methyl-cis-15-tetracosenoate (C-24:1 Δ^cis−15^) was done from Supelco (Bellefonte, PA, USA). Methanol, ethyl acetate, and hexane (chromatographic grade) were all obtained from Fisher Scientific (USA). Freshly prepared 10% H_2_SO_4_ in CH_3_OH was used, and purchasing of NaCl (analytical grade) was done from Sigma (St. Louis, MO, USA).

### Preparation of standard solutions

To prepare standard stock solutions I, 5 mg of each compound (total 15 standard solutions I) was dissolved in 1 mL hexane at room temperature. Twelve different levels of standard solutions for calibration were prepared for each FAME from the stock standard solution I having a concentration range of 2.5 mg/mL to 0.25 mg/mL using hexane for dilution. Deuterated myristic acid was used as internal standard. Until further analyzation all standard solutions were stored in −20 °C freezer.

### Sample preparation

FFAs extraction and GC-MS acquisition were done after random selection of samples. Aliquots (200 µL) of serum were spiked with an internal standard (IS) working solution (100 µL. deuterated myristic acid-d27 C14:0, 3.2 mg). Sample preparation was accomplished by the procedure proposed by Wang^21^.

### Gas chromatography-mass spectrometry (GC-MS)

GC (Agilent 7890) equipped with an autosampler, and coupled to a triple quadrupole mass spectrometer was used to perform GC-MS analysis. A capillary column having dimensions of ZB5MS, (Zeborn) 30 m × 0.25 mm I.D, and 0.25 µm film thickness was used to perform the separation. Helium was chosen as carrier gas; at the initial oven temperature, 9.8324 was the pressure of the carrier gas and flow rate was set to 1.20 mL min^−1^. Split mode (split/column flow ratio 50:1) was used for the injection of all standards and samples. Following optimized temperature program was used for separation of FAMEs at constant flow with (1) 50 °C for 2 minutes; (2) increasing at a rate of 7 °C per minute (3) 250 °C for 15 minutes. Electron impact (EI) at 70 eV having a scan range of 40–450 m/z was the mass spectrometer mode. Ion source and transfer line temperature was set to a value of 250 °C. 1 µL volume of sample was injected. For data acquisition and processing, Agilent Mass Hunter software was used. For quantification of FAMEs MRM method was used having a solvent delay of 5 min and collision energy of 5–45 eV. The scan rate was set to a value of 200 Cycle/ms and dwell time at 50 ms. Quantification was done by using fragment ions (listed in Table 1), one among them as quantifier and others as qualifiers. Separation of all fifteen FAMEs was achieved in 45 minutes on above stated optimized parameters giving a satisfactory resolution between peaks that include eight saturated FAMEs i.e. C-8:0, C-10:0, C-12:0, C-14:0, C-16:0, C-18:0, C-22:0 and C-24:0, seven monounsaturated FAMEs i.e. C-14:1 Δ^cis−9^, C-16:1 Δ^cis−9^, C-18:1Δ^cis−9^, C-18:1 Δ^cis−11^, C-20:1 Δ^cis−11^, C-22:1 Δ^cis−13^ and C-24:1 Δ^cis−15^ (Fig. S1). A good separation was also obtained in regioisomers i.e. methyl-cis-9-octadecenoate and methyl-cis-11-octadecenoate.

**Table 1:** Optimized GC-MS/MS acquisition method parameters and the list of precursor ions and product ions of each FAME.

FAMEs	Precursor Ion (m/z)	Optimized collision energy (eV)	MRM transitions (m/z)	
			Identification (q)	Quantification (Q)
C-8:0	158.2	30	41, 45, 55	158.2 → 73.0
C-10:0	186.2	15	59, 73, 115	186.2 → 101.0
C-12:0	214.3	15	69, 73, 83	214.3 → 101.0
C-14:0	242.4	15	69, 143, 157	242.4 → 101.0
C-14:1 Δ^cis−9^	208.0	10	84, 111, 137	208.0 → 97.0
C-16:0	270.4	10	171, 185, 199	270.4 → 101.0
C-16:1 Δ^cis−9^	236.0	10	84, 111, 137	236.0 → 97.0
C-18:0	298.5	15	69, 143, 199	298.5 → 101.0
C-18:1 Δ^cis−9^	296.4	20	81, 95, 101	296.4 → 101.0
C-18:1 Δ^cis−11^	296.4	10	127, 155, 213	296.4 → 141.0
C-20:1 Δ^cis−11^	292.0	10	111, 137, 249	292.0 → 98.0
C-22:0	354.6	10	101, 199, 213	354.6 → 101.0
C-22:1 Δ^cis−13^	320.0	10	111, 263, 277	320.0 → 98.0
C-24:0	382.6	10	157, 213, 227	382.6 → 101.0
C-24:1 Δ^cis−15^	348.0	10	69, 97, 123	348.0 → 97.0

Validation of developed method was achieved by various parameters including accuracy, regression coefficient, precision, limit of quantification and limit of detection. Standard solutions were used to determine precision and accuracy. Percent relative standard deviation (RSD) was used to express precision of the method from the calibration samples. The calibration curve was used for determination of limit of detection (LOD) and limit of quantification (LOQ). The calculation of LOD and LOQ was based on 3.3α/S and 10α/S, respectively; where ‘S’ is the slope of regression line and ‘α’ is the residual standard deviation of the y-intercept.

A quality control (QC) sample obtained from the pooling of serum of AL patients and controls, theoretically identical to the biological samples. For metabolomics study QC having same sample matrix and metabolic composition as biological samples is necessary^22^. QC samples were injected every after four serum samples in order to keep a check on the GC-MS condition in our study.

### Statistical analysis

Statistical analysis was carried out using RF to formulate a prediction and classification model to examine the alteration in FFAs profiles of the groups of our study. Among a variety of machine learning methods, a wide acceptance has been gained by RF, trees ensemble technique attributed by some advantages^23–25^. The detail RF modeling process can be found in Breiman’s study^26,27^.

## Results

### Method optimization and validation

Optimization of MRM method for quantification of FAMEs by GC-MS/MS was achieved by the following manner: (1) firstly, the retention time of each FAME was determined (2) selection of primary precursor ions, (3) optimization of the collision energy, and (4) establishment of MRM transitions. The selection of precursor ions is mainly based on selectivity instead of signal intensity. Homologous series of FAMEs differ by only fourteen mass units and produce nearly identical fragments. For that reason, molecular ions were used as precursor ions. An intense precursor peak or molecular peak was observed from saturated FAMEs in comparison to unsaturated FAMEs. These precursor ions were scanned for the product ions formed under different collision energies varying from 5 to 40 eV and 5–30 eV was the most suitable collision energies to obtain fragment ions with significant abundance. For all FAMEs, representative fragment ions and optimized collision energy are mentioned in Table 1. Critical analyzation of product ion spectra give assistance to choose three product ions with high responses, and for quantification high abundant product ions among them were used, on the other side, lower abundant product ions were chosen as qualifiers. The conformation of the compound was done by the ratio between these two ions. Figure S2 presented the spectra and chromatograms (quantifier and qualifiers) of all FAMEs.

Evaluation of various parameters was performed for validation of the method. These include accuracy, regression coefficient, linear range (LR), precision, LOQ and LOD. The relationship between the corresponding peak area and the concentration was found to be linear up to 2.5–0.25 mg mL^−1^ of concentration range for all FAMEs with a coefficient (r^2^) value of ≥0.95. Table 2 compiled the retention time, regression equation, calibration curve, and LR of each compound. FAMEs standards utilized for the estimation of accuracy and the value of accuracy was within the range of 78–116% in all FAMEs (Table S2). Percent relative standard deviation (% R.S.D) of triplicate (n = 6) analysis was performed to express the precision/repeatability of the method. In the analysis of all FAMEs, no significant variations (≤5%) were observed. Table 2 listed the LOD and LOQ of all saturated and unsaturated FAMEs that were analyzed by GC-MS/MS having values varied from 0.157–0.796 mg mL^−1^. Selection of adequate precursor and product ions was made to monitor appropriate MS-MS transitions of each FAME to increase the selectivity of the GC-MS/MS procedure.

**Table 2:** Retention time, correlation coefficients, regression equation, limits of detection and limit of quantification of individual FAMEs.

FAMEs	Retention time (min)	Correlation coefficient (r^2^)	Regression equation	LOD (mg mL^−1^)	LOQ (mg mL^−1^)
C-8:0	11.906 ± 0.003	0.9700	y = 5734.40* × −3456.13	0.2290	0.6939
C-10:0	15.959 ± 0.002	0.9804	y = 44689.78* × −26572.41	0.2375	0.7198
C-12:0	19.566 ± 0.003	0.9757	y = 13344.42* × −78077.45	0.2476	0.7504
C-14:0	22.806 ± 0.007	0.9839	y = 252579.39* × −162940.73	0.2272	0.6885
C-14:1 Δ^cis−9^	22.626 ± 0.005	0.9824	y = 186207.96* × −120464.25	0.2527	0.7658
C-16:0	25.743 ± 0.009	0.9865	y = 157066.81* × −103189.90	0.2308	0.6993
C-16:1 Δ^cis−9^	25.466 ± 0.007	0.9871	y = 114942.54* × −74618.51	0.2304	0.6983
C-18:0	30.590 ± 0.005	0.9837	y = 263816.71* × −152409.85	0.2440	0.7024
C-18:1 Δ^cis−9^	28.110 ± 0.009	0.9826	y = 533.64* × −318.89	0.2551	0.7732
C-18:1 Δ^cis−11^	28.176 ± 0.009	0.9581	y = 14400.43* × −8854.33	0.2423	0.7344
C-20:1 Δ^cis−11^	32.590 ± 0.005	0.9807	y = 63310.813* × −39123.02	0.1962	0.5948
C-22:0	33.90 ± 0.02	0.9834	y = 176081.82* × −114211.45	0.2628	0.7965
C-22:1 Δ^cis−13^	33.46 ± 0.01	0.9910	y = 66450.90* × −44433.49	0.1807	0.5476
C-24:0	38.53 ± 0.05	0.9766	y = 150162.93* × −103479.79	0.1577	0.4781
C-24:1 Δ^cis−15^	37.82 ± 0.02	0.9789	y = 34888.70* × −20911.09	0.2333	0.6999

### Chemometric analysis

Table 3 constitutes the qualitative and quantitative results of serum FFAs for ALL, AML, APA, MDS and healthy control that are obtained under the optimized conditions. The concentration of two of them lies below quantification limit which includes C-22:0 and C-16:1Δ^cis−9^. As could be seen in Table 3 the serum FFAs concentration of some of them differs remarkably among the groups. Figure S3 is the graphical representation that gives a summary of mean concentration of FFAs along with the standard deviation and range. Moreover, a pattern recognition analysis was done using RF to see whether these FFAs may perhaps separate AL groups from controls and which FFA could be used for group differentiation.

**Table 3:** FFAs composition of the HC, ALL, AML, APA and MDS samples.

FFAs (mg mL^−1^)	HC (n = 30)	ALL (n = 24)	AML (n = 62)	APA (n = 46)	MDS (n = 17)
C-8:0	0.699 ± 0.005	0.699 ± 0.003	0.702 ± 0.003	0.701 ± 0.002	0.701 ± 0.001
C-10:0	0.72 ± 0.03	0.724 ± 0.005	0.725 ± 0.005	0.721 ± 0.006	0.720 ± 0.003
C-12:0	0.758 ± 0.004	0.761 ± 0.005	0.76 ± 0.02	0.76 ± 0.02	0.75 ± 0.02
C-14:0	1.0 ± 0.2	0.80 ± 0.09	0.72 ± 0.04	0.8 ± 0.1	0.78 ± 0.06
C-14:1 Δ^cis−9^	0.77 ± 0.03	0.77 ± 0.01	0.77 ± 0.03	0.76 ± 0.03	0.77 ± 0.02
C-16:0	0.76 ± 0.06	0.8 ± 0.2	0.82 ± 0.07	0.8 ± 0.2	0.80 ± 0.04
C-16:1 Δ^cis^-^9^	*BQL	0.70 ± 0.05	*BQL	*BQL	*BQL
C-18:0	1.1 ± 0.4	0.78 ± 0.08	0.78 ± 0.07	0.81 ± 0.05	0.81 ± 0.06
C-18:1 Δ^cis−9^	0.77 ± 0.08	0.78 ± 0.1	0.8 ± 0.1	0.77 ± 0.07	0.78 ± 0.09
C-18:1 Δ^cis−11^	0.73 ± 0.07	0.74 ± 0.07	0.74 ± 0.09	0.74 ± 0.07	0.74 ± 0.06
C-20:1 Δ^cis−11^	0.65 ± 0.04	0.62 ± 0.04	0.62 ± 0.05	0.62 ± 0.05	0.61 ± 0.03
C-22:0	*BQL	*BQL	*BQL	*BQL	*BQL
C-22:1 Δ^cis−13^	0.675 ± 0.004	0.680 ± 0.008	0.672 ± 0.006	0.670 ± 0.007	0.6692 ± 0.0009
C-24:0	0.69 ± 0.04	0.69 ± 0.03	0.68 ± 0.04	0.68 ± 0.07	0.68 ± 0.02
C-24:1 Δ^cis−15^	0.7 ± 0.1	0.70 ± 0.07	0.70 ± 0.07	0.70 ± 0.07	0.70 ± 0.07

^*^Below Quantification Limit.

**Figure 1:**
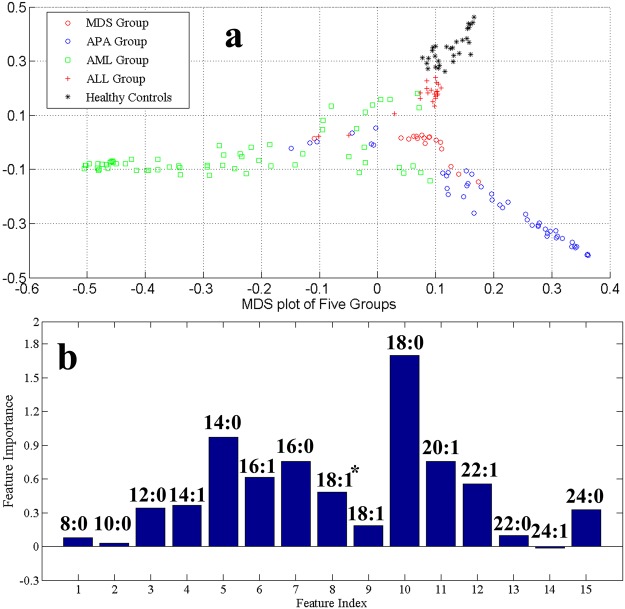
Serum FFAs profiles from healthy controls, ALL, AML, APA and MDS groups (**a**) Multi-dimension scaling plot (**b**) VIP plot obtained by RF model (^*^C-18:1Δ^cis−9^).

**Figure 2:**
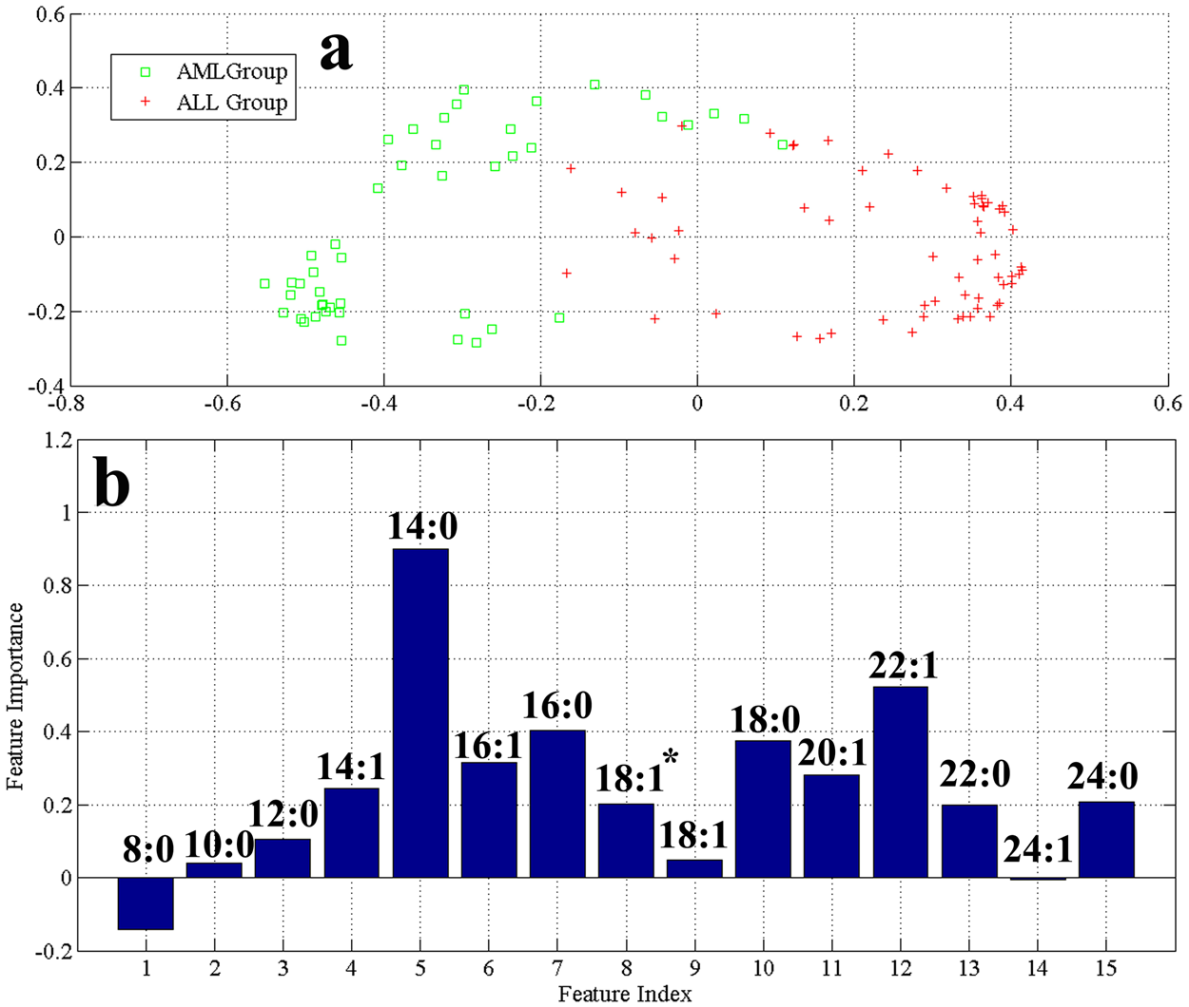
Serum FFAs profiles from ALL, AML groups (**a**) Multi-dimension scaling plot (**b**) VIP plot obtained by RF model (^*^C-18:1Δ^cis−9^).

**Figure 3:**
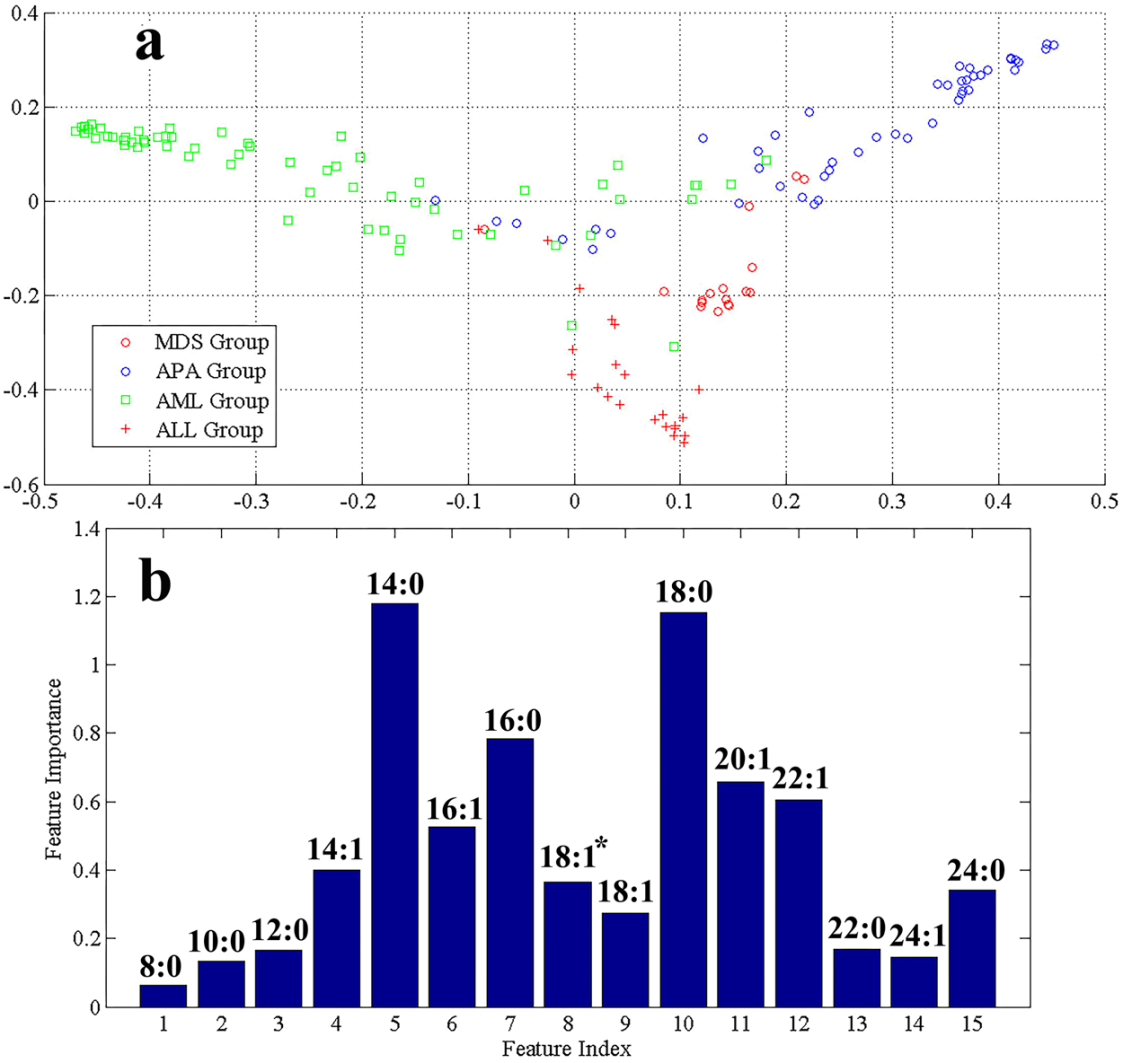
Serum FFAs profiles from ALL, APA, AML and MDS (**a**) Multi dimension scaling plot (**b**) VIP obtained by RF models (^*^C-18:1Δ^cis−9^).

The multi-dimension scaling plot of serum FFAs profiles from HC, ALL, APA, AML and MDS is shown in Fig. 1a. The cluster of HC samples appears separately in multi-dimension scaling plot. Likewise, a clear and better separation was observed for all other groups. The generated RF model using out-of-bag (OOB) estimation gave an overall accuracy of 96.6%, 96.0% sensitivity, and 96.5% specificity. These values suggested the effectiveness of RF model algorithm to imitate distinctive FFAs profiles for discrimination of different groups under consideration. Similarly, the multi-dimension scaling plots of serum FFAs profiles of AML and ALL (Fig. 2a) suggested that the respective groups are clustered separately with few overlap samples. Moreover, to find association of AL subgroups with pre-leukaemic controls, a multi-dimension scaling plot was generated (Fig. 3a) that reflects MDS, APA, and AML disease states have a significant trend of clustering together while ALL group appear separately using prediction ability of RF model with 82.7% sensitivity, 77.4% specificity and 82.7% accuracy.

**Figure 4:**
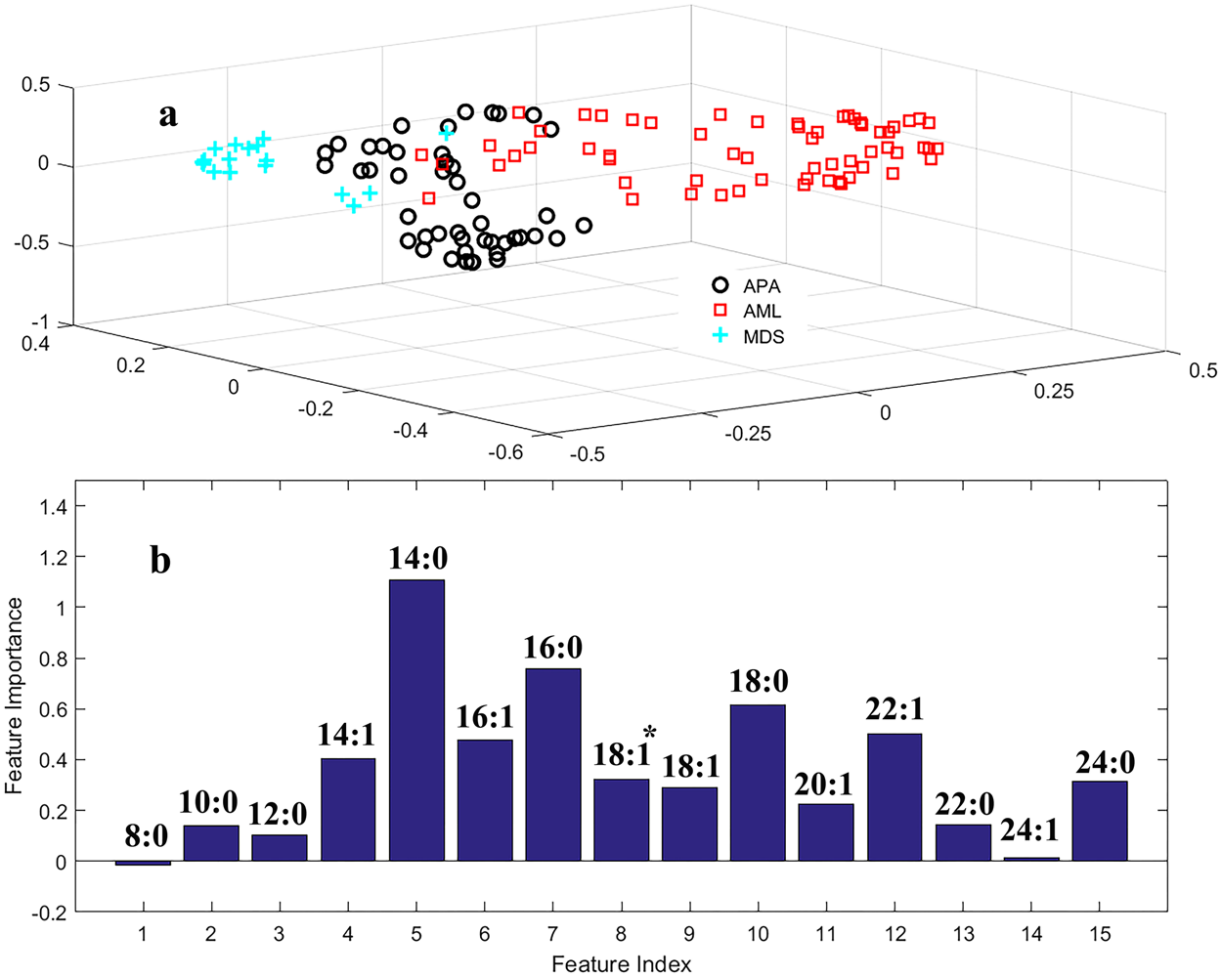
Serum FFAs profiles from APA and AML/MDS (**a**) Multi dimension scaling plot (**b**) VIP obtained by RF models (^*^C-18:1Δ^cis−9^).

To insight more into the association of APA with MDS/AML, the classification plot of APA and MDS/AML was generated (Fig. 4a). It can be clearly observed that according to FFA profile, APA lie in middle of MDS and AML groups, while three groups have some overlap areas, which also supports that APA patients can progress to both either MDS or AML under certain set of conditions. Moreover, it is worthy to notify that based on FFAs profile, MDS and AML groups locate in two different directions, these results suggested that involvement of fatty acids in the pathogenesis of MDS and AML is different. Therefore, these findings of FFAs might play an important role in understanding and identifying progression of pre-leukaemic states to AML in future.

A set of quantitative clinical parameters that are used for the diagnoses of AL and pre- leukaemic conditions, among them, four parameters were used for correlation analysis i.e. hemoglobin (Hb), total leukocyte count, neutrophil and absolute neutrophil count. To find the association between FFAs and the four clinical parameters, a canonical correlation analysis (CCA) was performed that classically based on correlation of linear relationships between two sets of multidimensional variables. Figure S4 represent the scatter plot of FFAs and clinical parameters and R value represents canonical correlation coefficient between clinical parameters and FFAs. As shown in the Fig. S4 clinical parameters and FFAs have a good correlation. The R value is 0.758, which reflect the FFAs in leukaemic and pre-leukaemic diseases effectively correlate with these clinical parameters and can contribute in disease diagnosis in future.

### Discerning of FFAs

Statistical evaluations of significant variables i.e. variable importance plot (VIP) having highest values were done through t-test. From the VIP (Fig. 1b), we found that compound **10** that is stearic acid have the highest contribution in classification among HC, ALL, AML, APA, and MDS with *p*-value < 0.05. It was worth noting that the results obtained in the classification plot Fig. 2a seemed to show a good discrimination between AML and ALL. In the VIP between AML and ALL (Fig. 2b), compound **5** i.e. C-14:0 (myristic acid) is found to be the potential element in differentiating between the two disease states having a *p*-value of 0.0038.

**Figure 5:**
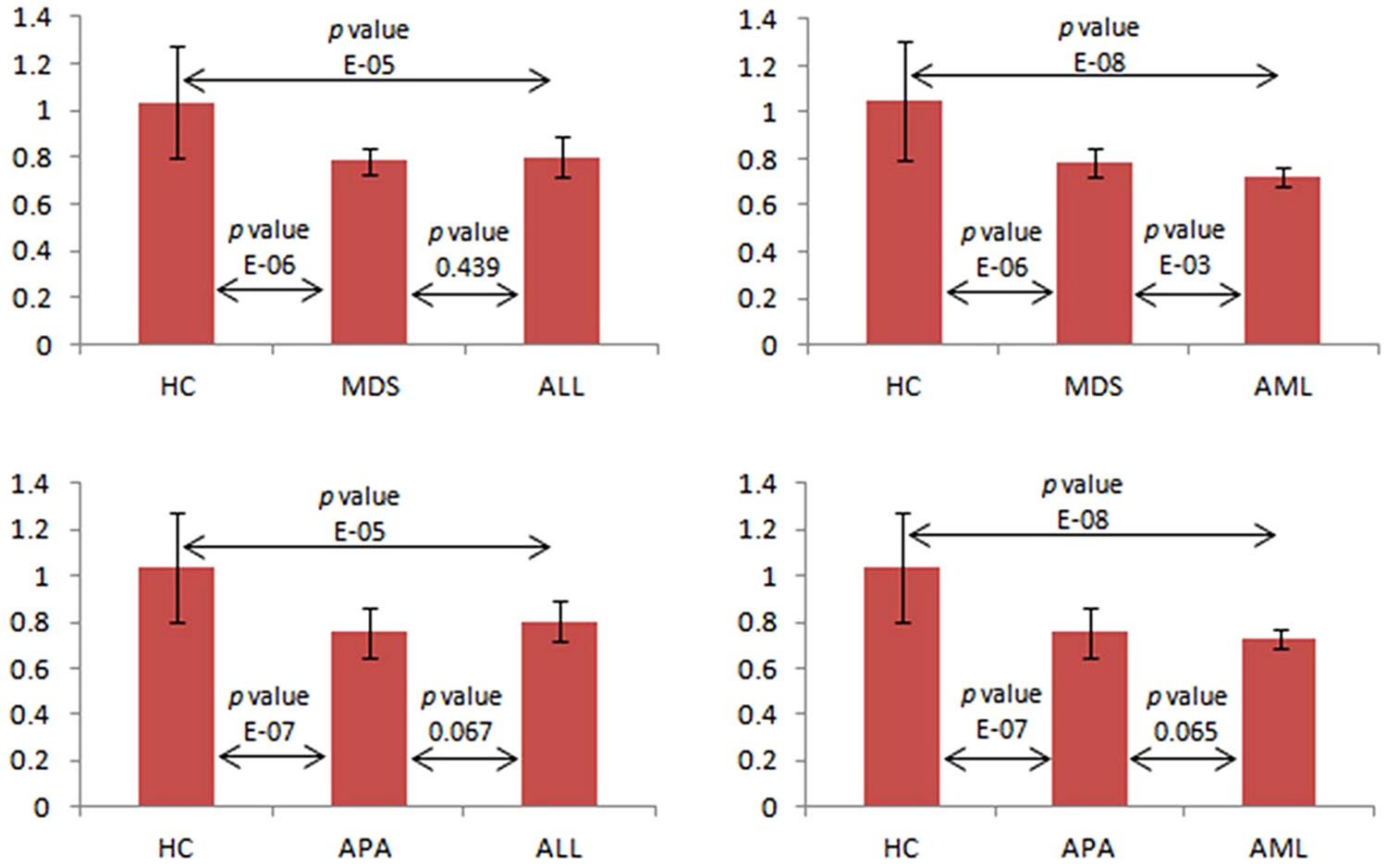
Mean concentration plot of C-14:0 in AL in comparison with pre-leukaemic diseases and healthy control.

For further evaluation of correlated FFAs between AL (ALL and AML) and pre-leukaemic conditions (APA and MDS), VIP plot was constructed for all disease groups (Fig. 3b). Figure 3b reflect that myristic acid (C-14:0) and stearic acid (18:0) found be the differentiative elements among AL (ALL and AML) and pre-leukaemic diseases (MDS and APA). To insight more into contribution of each FFAs in monitoring the progression of APA into MDS/AML a VIP plot was also performed (Fig. 4b). It was worth noting that compounds **5** and **7** (myristic acid and palmitic acid) again showed higher feature importance than other FFAs in progression of APA into MDS and AML. Therefore, it could be stated that these compounds may serve as progressive marker in monitoring progression of APA into MDS and AML. Compound **5** i.e. C-14:0 showed little higher VIP value, so further screened separately. In Fig. 5, mean concentration of C-14:0 was presented along with *p*-values. This figure contributes to show an orderly progression of pre-leukaemic conditions towards AML.

## Discussion

Abnormal cell growth and proliferation are the two main features of cancer, which needs energy, biosynthetic activity and certain nutrients or building blocks like proteins, lipids particularly FAs and nucleic acids to duplicate all macromolecular components in the course of each passage through the cell cycle. The metabolism of cancer cells is reprogrammed in order to accumulate metabolic intermediates as sources of these building blocks and to gain energy. An important metabolic hallmark of cancer is reprogramming of lipid metabolism. Among four lipid moieties [FAs, phospholipids, cholesterol and neutral triglycerides (TAG)], alteration of FAs in the blood stream is the most important aberration in cancer cell metabolism. Cancer cells show a strong FAs avidity by over activating their de novo synthesis and a shift towards FAs synthesis was observed^6^. Activation of some oncogene and expression of some proteins may also contribute to increase the levels of FAs in cancer. For example, fatty acid binding protein 4 (FABP4), a lipid chaperone, was observed in providing FAs from surrounding adipocytes for ovarian tumors^28^. Ultimately, these FAs are involved in providing macromolecules in membrane generation, proteins modification, bioenergetic pathways and in signaling during development and progression of cancer. Involvement of FAs in AL conditions also borne out by few past and the current examination^18,20,29^. Each of these studies report different set of FFAs in specific AL condition. Moreover, there are few reports that suggest different constructive roles of FFAs in leukaemia^30,31^. But still has a lack in comparison of AL FFAs profile with pre-leukaemic condition. Here we are the first to examine and quantify FFAs profile in AL and its pre-states.

Figures 1b, 2b, 3b and 4b reveal that compounds **5**, **7**, **10** (myristic acid, palmitic acid and stearic acid, respectively) have the highest contribution in the discrimination of respective groups. Stearic acid is found to be the key player in discriminating HC group from AL and pre-leukaemic states. It is a natural saturated fatty acid and a very important bioactive molecule found in human biofluids. As nutrients, it is not just the main energy source but additionally serves as a signaling molecule in a number of cellular processes. Due to the continuous higher concentration of stearic acid in plasma, it has a major role in lipotoxicity. Stearic acid may also influence the insulin sensitivity and substrate oxidation. It also works as a potential biomarker in benign liver tumor patients those with hepatitis and liver cirrhosis^32–34^. Different aspects of involvement of stearic acid in cancer have been reported like role of stearic acid in induction of apoptosis that prevent development cancer formation, on the other hand having a negative role in DNA damage that promote cell transformation and in turn tumorigenesis^35,36^. Other studies declare little or no effect of stearic acid in promoting tumor^37^. It was also observed that a reduction in the ratio of stearic acid and oleic acid may serve as an indication of malignancy^38–40^. Reports on AL lipodomics state a lower concentration of stearic acid in human blood which probably due to its oxidation, confirmed from the fact that FAO (fatty acid oxidation) is one of the crucial pathway in AL cells^41,42^.

Among all disease groups myristic acid attain the highest value to serve as progressive marker. As it is one among few natural saturated fatty acid and involves in the stabilization of many different proteins like proteins involved in the fight against tumor and in the immune system. This function is referred as myristoylation^43^. It is estimated that in human about 0.5% proteins are myristoylated. In addition, this acid has effects on blood coagulation, insulin resistance, blood cholesterol and inflammation^1^. All these factors are also linked to AL. A former study showed the downturn concentration of myristic acid found in peripheral blood aspirates compared to the peripheral blood, suggesting a higher consumption of these FFAs by malignant lymphoblasts^20^. This is an indication of the fact that cancer cells consumed myristic acid for their rapid proliferation. In addition to myristic acid another saturated fatty acid i.e. palmitic acid predominantly found attached to proteins in eukaryotic cells^44^ and its involvement in leukaemia and pre leukaemia has also been identified in our present study. Involvement of palmitic acid in many metabolic pathway and alteration in its level in blood was also reported in many diseases like breast cancer, colorectal cancer^45^. It also involved in boosting of metastasis in human oral cancer cells and has the strongest effect in this boosting among all fatty acids. A high concentration of this acid in biofluid increase this process and spreading of cancer^46^. Other reports declare palmitic acid inductive role in apoptosis and negative inhibitory role in DNA damage^35,36^, both of these factors are directly related the promotion of cancer progression.

## Conclusion

This study provides a comparative analysis of the selected FFAs in controls (healthy and pre-leukaemic) with AL patients. The results proposed that metabolism of stearic acid (18:0) was found to be differentiated among healthy, pre-leukaemic and AL states. Furthermore, the result reflects that myristic acid (14:0) could be used as a discriminative marker between AML and ALL. Additionally, myristic acid (14:0) may serve as a differentiative marker of pre-leukaemic (APA and MDS) into AL. Combination of myristic acid (C 14:0) and palmitic acid (16:0) can also be used to monitor progression of APA into MDS or AML, after validation of above results over a large set of samples. Moreover, GC-MRM-MS in combination with RF algorithm may serve as a powerful and efficient strategy to explore metabolic fingerprints and perturbations of serum FFAs and provide potential biomarkers in other experimental design too. Overall, this study demonstrated that FAs metabolism not only altered in AL but also in pre-leukaemic diseases (i.e. MDS, APA). Therefore, profile of these differentiating FAs can be used in future to identify progression of pre- leukaemia to AL.

## Electronic supplementary material

Supplementary Information
