# Supplementary material for: Alteration of Serum Free Fatty Acids are Indicators for Progression of Pre-leukaemia Diseases to Leukaemia
Source: Sci Rep. 2018 Oct 5;8:14883. doi: 10.1038/s41598-018-33224-1 (PMC6173776; doi:10.1038/s41598-018-33224-1)
Supplement: Supplementary file 1 — Supplementary Information [file 41598_2018_33224_MOESM1_ESM.pdf]

# Alteration of Serum Free Fatty Acids are Indicators for Progression of Pre-leukaemia Diseases to Leukaemia

Ayesha Khalid[a], Amna Jabbar Siddiqui[a], Jian-Hua Huang[d], Tahir Shamsi[c], Syed Ghulam Musharraf[ab*]

[a]*H.E.J. Research Institute of Chemistry, International Center for Chemical and Biological Sciences, University of Karachi, Karachi-75270, Pakistan*

[b]*Dr. Panjwani Center for Molecular Medicine and Drug Research, International Center for Chemical and Biological Sciences, University of Karachi, Karachi-75270, Pakistan*

[c]*National Institute of Blood Diseases and Bone Marrow Transplantation, Karachi, Pakistan*

[d]*TCM and Ethnomedicine Innovation and Development Laboratory, Changsha,Hunan,China*

*Corresponding author. Tel.: +92 213 4824924-5; 4819010; fax: + 92 213 4819018-9.

*E-mail address:* musharraf1977@yahoo.com

**Supplementary Table S1:** Clinical classification of patients.

| | Subtypes | Number of patients |
|---|---|---|
| **AML** | Acute panmyelosis with myelofibrosis | 1 |
| | AML without maturation | 16 |
| | AML with maturation | 22 |
| | Acute promyelocytic leukemia | 12 |
| | Acute myelomonocytic leukemia | 4 |
| | Acute monoblastic leukemia | 2 |
| | Acute monocytic leukemia | 3 |
| | not classified at the time of sampling | 4 |
| **ALL** | B-cell | 3 |
| | CALLA positive | 4 |
| | CALLA positive and MPO negative | 2 |
| | MPO negative | 35 |
| | precursor B cell | 11 |
| | T cell | 12 |
| | not classified at the time of sampling | 5 |

CALLA: common acute lymphoblastic leukemia-associated antigen

MPO: Myeloperoxidase enzyme

**Table S2**: Accuracy and precision data for all FAME standards.

| Compounds | Concentration (mg ml$^{-1}$) | Found (mg ml$^{-1}$) | RSD (%) | Accuracy (%) |
|---|---|---|---|---|
| | 1.310 | 1.034±0.01 | 1.310 | 78.952 |
| C-8:0 | 1.048 | 1.06±0.06 | 1.048 | 101.114 |
| | 5.000 | 5.82±0.02 | 5.00 | 116.437 |
| | 1.310 | 1.221±0.02 | 1.645 | 93.191 |
| C-10:0 | 1.048 | 1.017±0.007 | 0.768 | 97.003 |
| | 5.000 | 5.54±0.05 | 0.739 | 110.386 |
| | 1.310 | 1.34±0.02 | 1.732 | 102.002 |
| C-12:0 | 1.048 | 1.07±0.01 | 1.174 | 100.586 |
| | 5.000 | 5.38±0.06 | 1.082 | 107.685 |
| | 1.310 | 1.3±0.1 | 1.537 | 102.377 |
| C-14:0 | 1.048 | 1.08±0.02 | 1.831 | 102.666 |
| | 5.000 | 5.35±0.02 | 2.524 | 106.974 |
| | 1.310 | 1.32 ±0.02 | 1.410 | 100.943 |
| C-16:0 | 1.048 | 1.07±0.01 | 1.382 | 101.943 |
| | 5.000 | 5.3±0.1 | 2.339 | 107.057 |
| | 1.310 | 1.35±0.02 | 1.840 | 102.997 |
| C-16:1 $\Delta^{cis-9}$ | 1.048 | 1.10±0.01 | 1.413 | 104.586 |
| | 5.000 | 5.3±0.1 | 2.207 | 107.034 |
| | 1.310 | 1.37±0.02 | 1.383 | 104.825 |
| C-18:0 | 1.048 | 1.08±0.02 | 1.797 | 103.522 |
| | 5.000 | 5.25±0.05 | 0.988 | 105.047 |
| | 1.048 | 1.09±0.03 | 3.288 | 103.652 |
| C-18:1 $\Delta^{cis-9}$ | 1.638 | 1.51 ±0.01 | 0.8202 | 92.273 |
| | 5.000 | 5.2 ±0.1 | 2.508 | 103.776 |
| | 1.310 | 1.32±0.01 | 1.204 | 101.031 |
| C-22:0 | 1.048 | 1.07±0.01 | 1.298 | 101.887 |
| | 5.000 | 5.3±0.1 | 2.193 | 106.972 |
| | 1.310 | 1.36±0.03 | 2.020 | 103.738 |
| C-22:1 $\Delta^{cis-13}$ | 1.048 | 1.084±0.007 | 0.686 | 103.391 |
| | 5.000 | 5.31±0.07 | 1.319 | 106.214 |
| | 1.310 | 1.35 ±0.02 | 1.893 | 102.976 |
| C-24:0 | 1.048 | 1.08±0.01 | 0.988 | 102.920 |
| | 5.000 | 5.3 ±0.1 | 2.560 | 105.899 |
| | 1.31 | 1.35 ±0.03 | 2.601 | 104.812 |
| C-24:1 $\Delta^{cis-15}$ | 1.048 | 1.08±0.02 | 1.662 | 102.980 |
| | 5.000 | 5.3 ±0.1 | 2.425 | 106.250 |

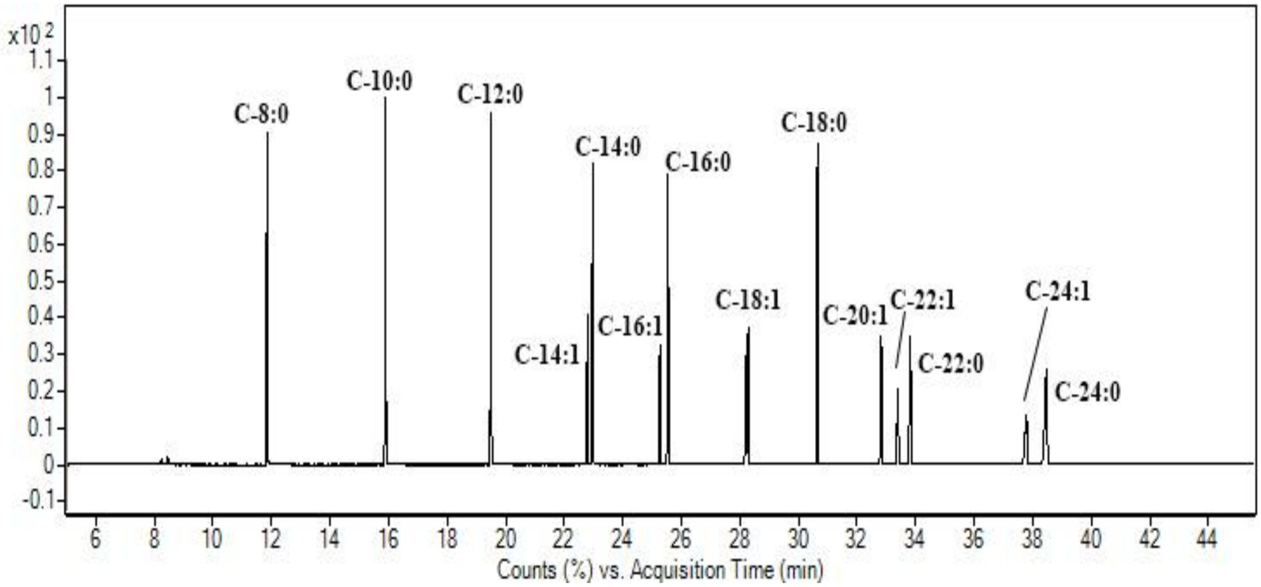


**Figure S1:** Full positive TIC chromatogram of FAME standards.

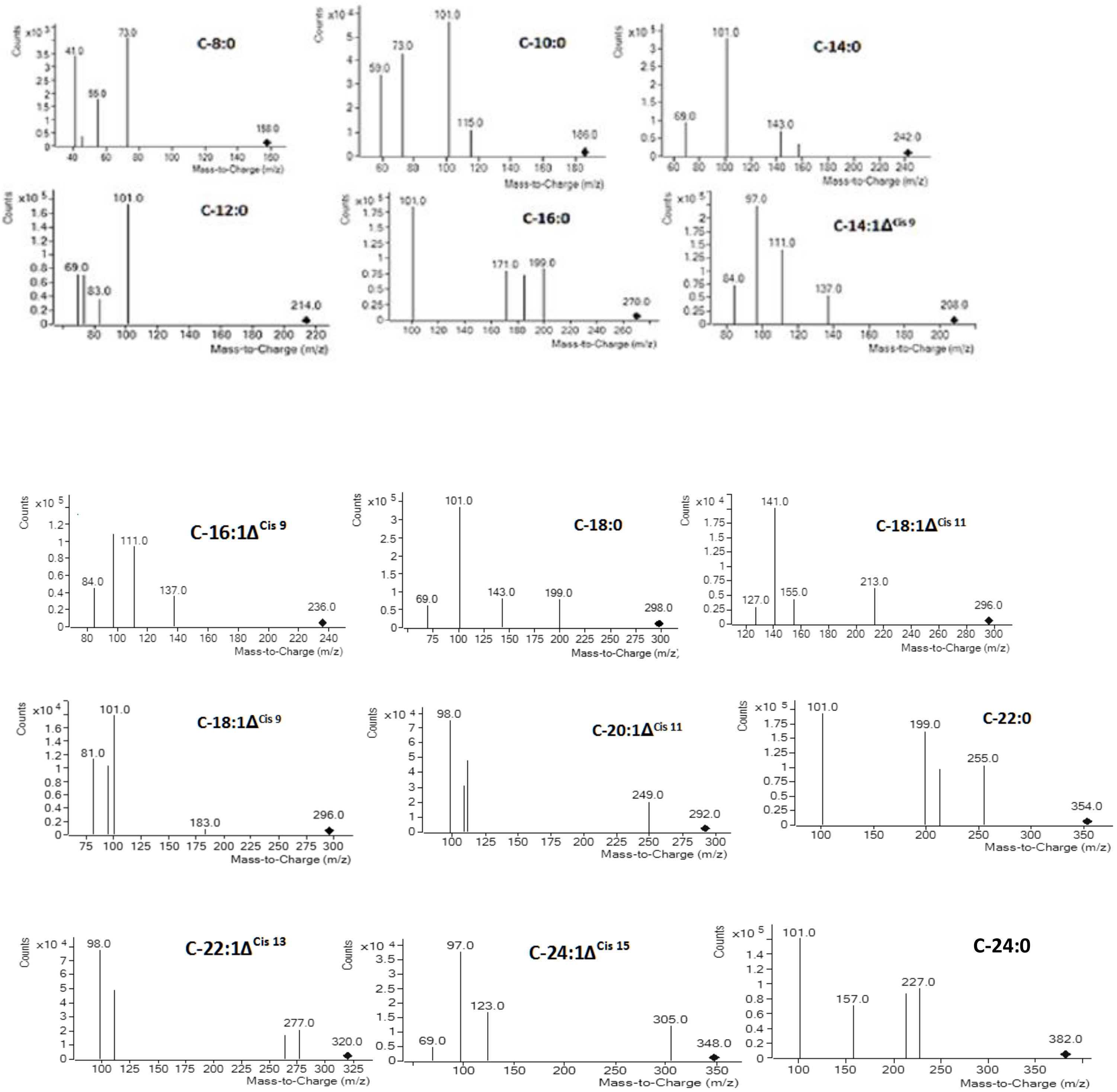


**Figure S2**: Spectra of quantifer and qualifiers of all FAMEs.

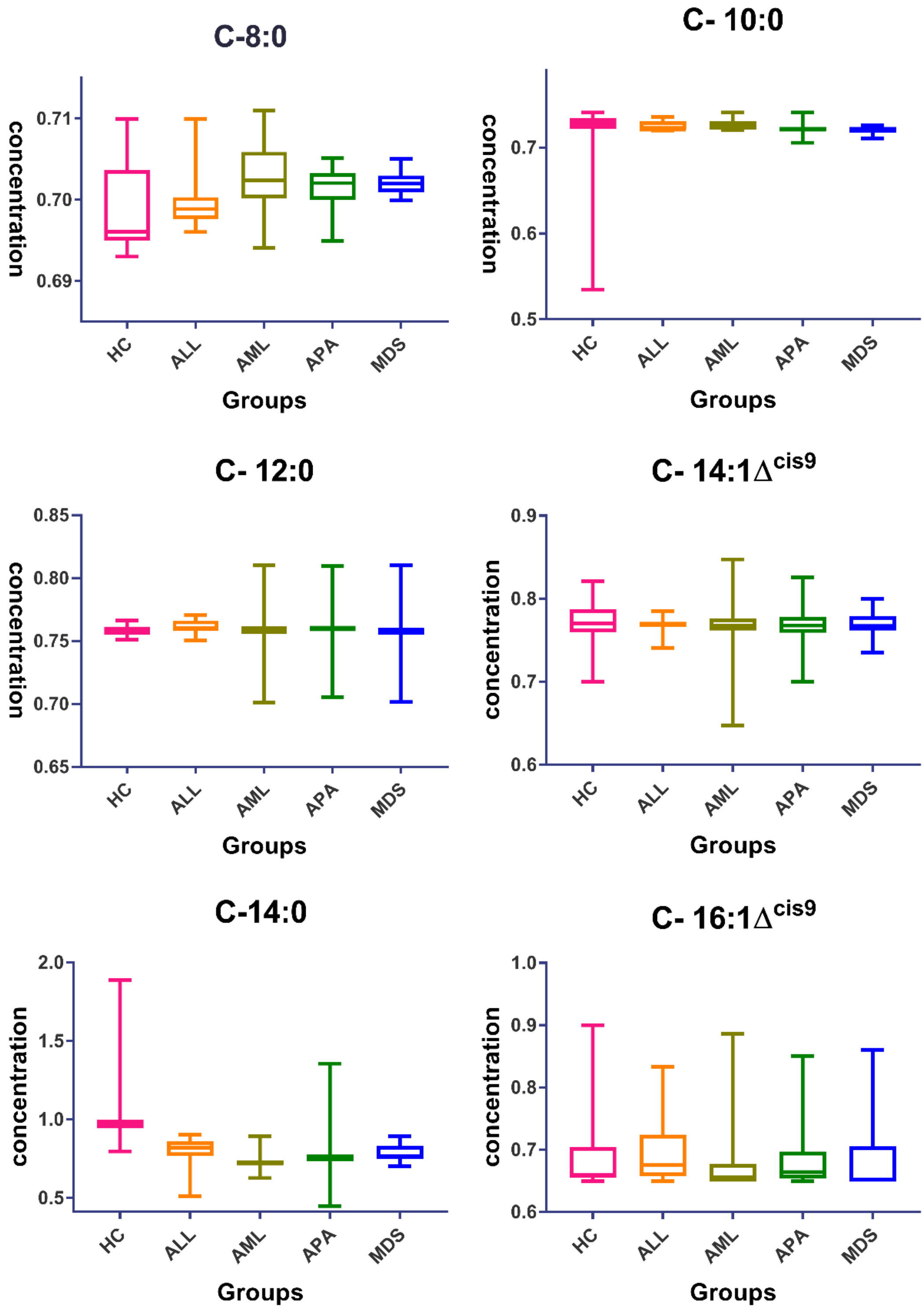

C-8:0
concentration
0.71
0.70
0.69
HC
ALL
AML
APA
MDS
Groups
C- 10:0
concentration
0.7
0.6
0.5
HC
ALL
AML
APA
MDS
Groups
C- 12:0
concentration
0.85
0.80
0.75
0.70
0.65
HC
ALL
AML
APA
MDS
Groups
C- 14:1Δcis9
concentration
0.9
0.8
0.7
0.6
HC
ALL
AML
APA
MDS
Groups
C-14:0
concentration
2.0
1.5
1.0
0.5
HC
ALL
AML
APA
MDS
Groups
C- 16:1Δcis9
concentration
1.0
0.9
0.8
0.7
0.6
HC
ALL
AML
APA
MDS
Groups

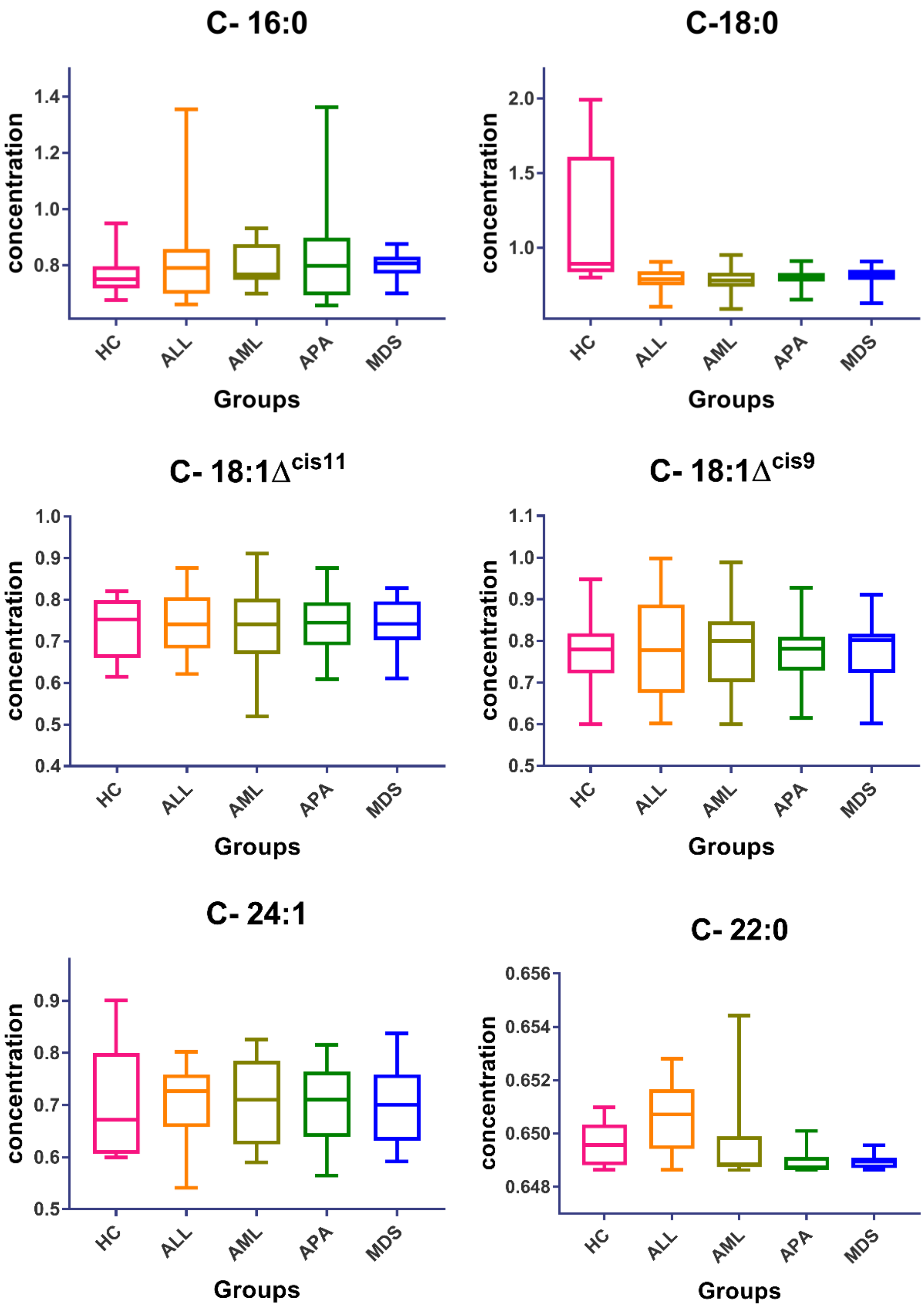
C- 16:0
concentration
1.4
1.2
1.0
0.8
HC
ALL
AML
APA
MDS
Groups
C-18:0
concentration
2.0
1.5
1.0
HC
ALL
AML
APA
MDS
Groups
C- 18:1Δcis11
concentration
1.0
0.9
0.8
0.7
0.6
0.5
0.4
HC
ALL
AML
APA
MDS
Groups
C- 18:1Δcis9
concentration
1.1
1.0
0.9
0.8
0.7
0.6
0.5
HC
ALL
AML
APA
MDS
Groups
C- 24:1
concentration
0.9
0.8
0.7
0.6
0.5
HC
ALL
AML
APA
MDS
Groups
C- 22:0
concentration
0.656
0.654
0.652
0.650
0.648
HC
ALL
AML
APA
MDS
Groups

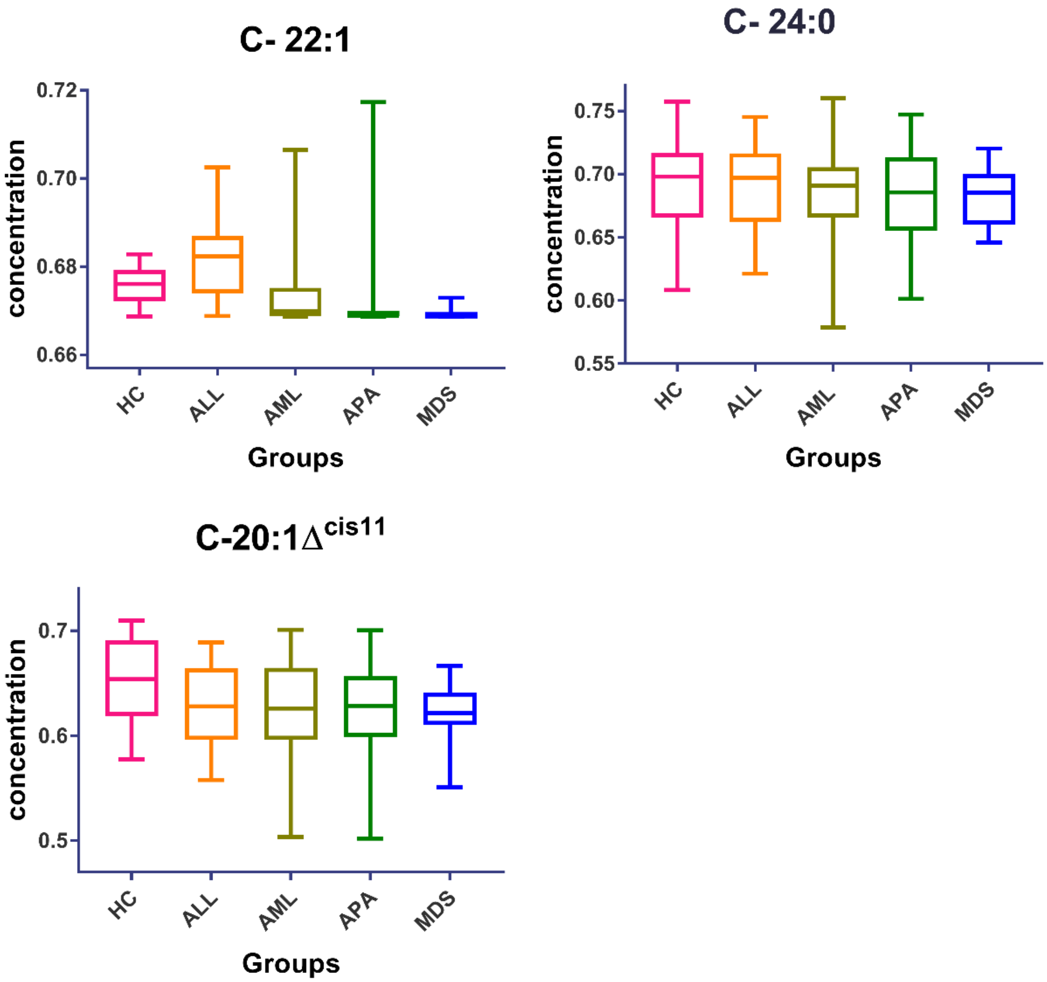


**Figure S3**: Box and whisker plot serum FFAs listed in table 3.

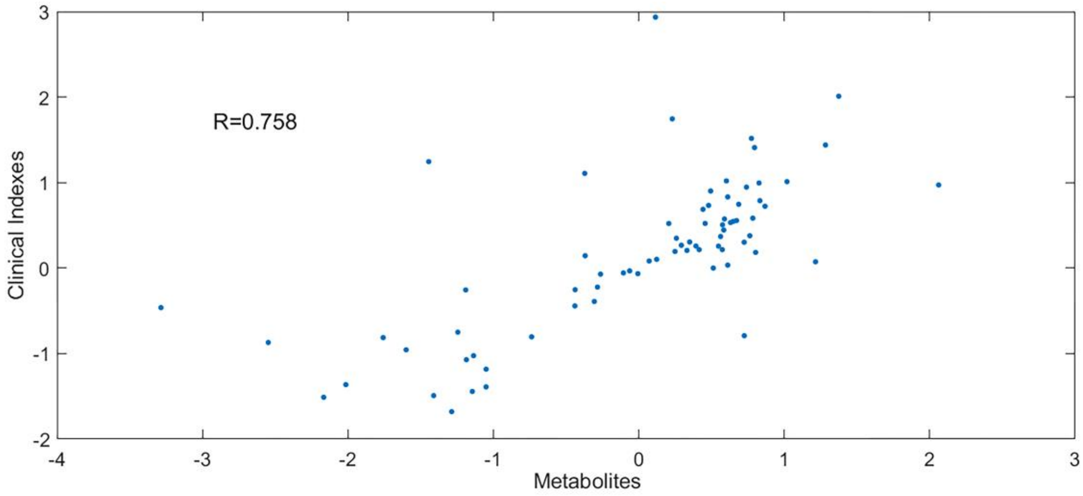


**Figure S4:** Canonical Variables scatter plot for serum FFAs and clinical parameters.
